# Supplementary material for: The Intersection of a Child’s Demographics and Household Socioeconomic Status in the Multimorbidity of Malaria, Anaemia, and Malnutrition among Children Aged 6–59 Months in Nigeria
Source: Int J Environ Res Public Health. 2024 May 19;21(5):645. doi: 10.3390/ijerph21050645 (PMC11120798; doi:10.3390/ijerph21050645)
Supplement: Supplementary file 1 [file ijerph-21-00645-s001.zip › ijerph-2959382-supplementary.pdf]

Supplementary file S1

In S1 (Model 3) shows that the proportional odds of children aged 24-35 months cohabiting with '2 or more diseases' versus combined of '0/1 disease' and living in the wealthiest households were significantly 0.66 lower than the odds for children aged 24-35 months and reside in poorest households when other covariates are held constant. Similarly, the proportional odds of children who are aged 34-47 months and live in the middle, richer, and richest household wealth quintiles were statistically significantly 0.59 (95% CI: 0.35-0.99), 0.49 (95% CI: 0.29-0.83), and 0.21 (95% CI: 0.12-0.36), respectively times the odds of contracting '2 or more diseases' versus combining of '0/1 disease' when compared with children aged 34-47 months and live in poorest household wealth quintile. Also, the two-way interactions between a child's age and wealth status reveal that children aged 48-59 months from the middle and richest classes have reduced statistically significantly proportional odds of 0.42 and 0.73, respectively, of being sick of '2 or more diseases' versus combined odds of '0/1 disease' when compared with children aged 48-59 months and live in the poorest household wealth quintile relative to the model 10 covariates.

However, model 4, which includes the covariates and all the possible two-way interactions between a child's sex, age, and wealth status, did not show any more improvement in the significant effects of the child's age and wealth status over what was attained in model 3. Furthermore, the three-way interaction between a child's sex, age and wealth status considered in model 5 did not yield any significant effects on children contracting '2 or more diseases' versus combining '0/1 disease' when compared with their respective reference categories conditional all the covariates in model 4.

S1: Multivariate analysis of Interactions between child's age, sex, and household wealth status accounting for other covariates

	Additive Model containing only the principal predictors (Child's sex, age and wealth status) N=7794			Child's sex * wealth status and all covariates N=7794			Child's sex* age and all covariates N=7794			Child's age* wealth status and all covariates N=7794			Child's sex * wealth status + child's sex * age + child's age * wealth status + all the covariates N=7794			Child's sex* age* wealth + all the covariates N=7794		
				Model 1			Model 2			Model 3			Model 4			Model 5		
Child's sex	AOR	p-value	95% CI	AOR	p-value	95% CI	AOR	p-value	95% CI	AOR	p-value	95% CI	AOR	p-value	95% CI	AOR	p-value	95% CI
Male	1.00			1.00			1.00			1.00			1.00			1.00		
Female	0.73	<0.001	(0.67-0.80)	0.66	0.001	(0.52-0.84)	0.70	0.005	(0.55-0.9)	0.73	<0.001	(0.67-0.8)	0.65	0.01	(0.47-0.9)	0.73	0.31	(0.4-1.35)
Child's age in group																		
6-11 months	1.00			1.00			1.00			1.00			1.00			1.00		
12-23 months	1.39	<0.001	(1.18-1.65)	1.39	<0.001	(1.17-1.65)	1.47	0.001	(1.17-1.85)	1.56	0.022	(1.07-2.28)	1.65	0.02	(1.08-2.51)	1.83	0.04	(1.03-3.24)
24-35 months	1.25	0.05	(1.00-1.57)	1.25	0.055	(0.99-1.56)	1.20	0.209	(0.9-1.59)	2.00	0.003	(1.27-3.18)	1.94	0.01	(1.18-3.18)	2.96	0.00	(1.45-6.04)
36-47 months	1.1	0.41	(0.88-1.38)	1.10	0.429	(0.87-1.38)	1.07	0.634	(0.81-1.42)	2.06	0.001	(1.33-3.18)	2.02	0.00	(1.26-3.25)	1.89	0.05	(1-3.57)
48-59 months	0.87	0.24	(0.69-1.10)	0.87	0.231	(0.69-1.09)	0.81	0.14	(0.61-1.07)	1.51	0.064	(0.98-2.34)	1.40	0.16	(0.87-2.24)	1.36	0.32	(0.74-2.52)
Household wealth index																		
Poorest	1.00			1.00			1.00			1.00			1.00			1.00		
Poorer	0.86	0.12	(0.72-1.04)	0.87	0.3	(0.68-1.13)	0.86	0.114	(0.72-1.04)	0.77	0.207	(0.51-1.16)	0.77	0.26	(0.49-1.21)	0.88	0.68	(0.47-1.63)
Middle	0.69	<0.001	(0.56-0.86)	0.63	0.001	(0.49-0.83)	0.69	0.001	(0.56-0.86)	0.97	0.874	(0.64-1.47)	0.88	0.58	(0.56-1.38)	0.93	0.81	(0.51-1.69)
Richer	0.56	<0.001	(0.44-0.72)	0.53	<0.001	(0.4-0.71)	0.56	<0.001	(0.44-0.71)	0.82	0.379	(0.53-1.27)	0.77	0.29	(0.48-1.24)	0.78	0.43	(0.42-1.44)
Richest	0.38	<0.001	(0.29-0.50)	0.34	<0.001	(0.24-0.47)	0.38	<0.001	(0.29-0.5)	0.94	0.802	(0.59-1.51)	0.82	0.45	(0.5-1.36)	0.97	0.92	(0.5-1.87)
**Interaction effects**																		
Sex and wealth status																		
				1.00									1.00			1.00		
Female and poorer				0.97	0.877	(0.7-1.35)							0.97	0.85	(0.7-1.35)	0.77	0.52	(0.34-1.74)
Female and middle				1.19	0.276	(0.87-1.62)							1.18	0.30	(0.86-1.61)	1.08	0.85	(0.49-2.37)
Female and richer				1.11	0.514	(0.81-1.51)							1.10	0.53	(0.81-1.5)	1.12	0.79	(0.51-2.45)
Female and richest				1.25	0.167	(0.91-1.72)							1.28	0.13	(0.93-1.76)	0.94	0.88	(0.41-2.16)
Sex and age																		
							1.00						1.00			1.00		
Female and 12-23 months							0.90	0.488	(0.66-1.22)				0.88	0.42	(0.65-1.2)	0.73	0.42	(0.34-1.57)
Female and 24-35 months							1.10	0.561	(0.8-1.52)				1.07	0.69	(0.77-1.48)	0.52	0.15	(0.21-1.27)
Female and 36-47 months							1.06	0.702	(0.77-1.46)				1.04	0.82	(0.75-1.43)	1.17	0.71	(0.52-2.64)
Female and 48-59 months							1.18	0.32	(0.85-1.62)				1.15	0.38	(0.84-1.58)	1.23	0.61	(0.55-2.76)
Age and wealth status																		
12-23 months and poorer										1.38	0.23	(0.82-2.32)	1.39	0.22	(0.82-2.33)	1.12	0.78	(0.52-2.4)
12-23 months and middle										0.9	0.67	(0.54-1.48)	0.91	0.70	(0.55-1.5)	0.86	0.70	(0.41-1.81)
12-23 months and richer										0.84	0.499	(0.51-1.39)	0.84	0.51	(0.51-1.39)	0.91	0.79	(0.44-1.87)
12-23 months and richest										0.63	0.08	(0.37-1.06)	0.64	0.09	(0.38-1.08)	0.44	0.04	(0.21-0.96)
24-35 months and poorer										1.08	0.80	(0.61-1.92)	1.08	0.78	(0.61-1.93)	0.65	0.35	(0.27-1.59)
24-35 months and middle										0.64	0.11	(0.37-1.1)	0.64	0.11	(0.37-1.11)	0.38	0.02	(0.16-0.88)
24-35 months and richer										0.61	0.078	(0.36-1.06)	0.62	0.08	(0.36-1.06)	0.42	0.04	(0.18-0.96)
24-35 months and richest										0.34	0.00	(0.19-0.6)	0.34	0.00	(0.19-0.6)	0.20	0.01	(0.08-0.48)
36-47 months and poorer										1.13	0.67	(0.65-1.94)	1.13	0.65	(0.66-1.95)	1.23	0.62	(0.54-2.81)

36-47 months and middle	*0.59*	*0.05*	*(0.35-0.99)*	*0.59*	*0.05*	*(0.35-0.99)*	*0.64*	*0.25*	*(0.29-1.37)*
36-47 months and richer	*0.49*	*0.007*	*(0.29-0.83)*	*0.49*	*0.01*	*(0.29-0.83)*	*0.50*	*0.08*	*(0.23-1.09)*
36-47 months and richest	*0.21*	*0.00*	*(0.12-0.36)*	*0.21*	*0.00*	*(0.12-0.36)*	*0.25*	*0.01*	*(0.11-0.56)*
48-59 months and poorer	*1.03*	*0.92*	*(0.6-1.77)*	*1.03*	*0.90*	*(0.6-1.78)*	*0.96*	*0.92*	*(0.44-2.12)*
48-59 months and middle	*0.58*	*0.04*	*(0.35-0.98)*	*0.59*	*0.05*	*(0.35-0.99)*	*0.67*	*0.29*	*(0.31-1.42)*
48-59 months and richer	*0.60*	*0.051*	*(0.36-1)*	*0.60*	*0.06*	*(0.36-1.01)*	*0.68*	*0.30*	*(0.32-1.42)*
48-59 months and richest	*0.27*	*0.00*	*(0.16-0.47)*	*0.27*	*0.00*	*(0.16-0.47)*	*0.25*	*0.01*	*(0.11-0.54)*
Sex, age and wealth status									
Female, 12-23 months and poorer							*1.50*	*0.44*	*(0.53-4.27)*
Female, 12-23 months and middle							*1.08*	*0.88*	*(0.4-2.96)*
Female, 12-23 months and richer							*0.82*	*0.69*	*(0.3-2.21)*
Female, 12-23 months and richest							*2.03*	*0.18*	*(0.72-5.74)*
Female, 24-35 months and poorer							*2.40*	*0.15*	*(0.74-7.78)*
Female, 24-35 months and middle							*2.47*	*0.11*	*(0.81-7.5)*
Female, 24-35 months and richer							*1.87*	*0.27*	*(0.62-5.65)*
Female, 24-35 months and richest							*2.49*	*0.12*	*(0.79-7.86)*
Female, 36-47 months and poorer							*0.87*	*0.81*	*(0.29-2.64)*
Female, 36-47 months and middle							*0.88*	*0.81*	*(0.31-2.51)*
Female, 36-47 months and richer							*0.96*	*0.94*	*(0.34-2.73)*
Female, 36-47 months and richest							*0.75*	*0.60*	*(0.25-2.25)*
Female, 48-59 months and poorer							*1.13*	*0.83*	*(0.38-3.36)*
Female, 48-59 months and middle							*0.76*	*0.61*	*(0.27-2.16)*
Female, 48-59 months and richer							*0.77*	*0.63*	*(0.27-2.18)*
Female, 48-59 months and richest							*1.21*	*0.73*	*(0.41-3.57)*
